# Pacemaker Implantation following Heart Transplantation: Analysis of a Nation-Wide Database

**DOI:** 10.3390/jcm12041604

**Published:** 2023-02-17

**Authors:** Ilias P. Doulamis, BoChang Wu, Armaan F. Akbar, Andreas Xanthopoulos, Emmanuel Androulakis, Alexandros Briasoulis

**Affiliations:** 1 Department of Surgery, Johns Hopkins University School of Medicine, Baltimore, MD 21287, USA; 2 Division of Cardiac Surgery, Department of Surgery, Johns Hopkins Hospital, Baltimore, MD 21287, USA; 3 Medical School of Athens, National and Kapodistrian University of Athens, 11527 Athens, Greece; 4 Department of Cardiology, St George’s University Hospital, London SW17 0QT, UK; 5 Division of Cardiovascular Medicine, Section of Heart failure and Transplantation, University of Iowa, Iowa City, IA 52242, USA

**Keywords:** pacemaker implantation, heart transplantation, registry, ECMO

## Abstract

Background: The 2018 United-Network-for-Organ-Sharing (UNOS) allocation-system changes resulted in greater recognition of mechanical circulatory support (MCS), leading to more heart transplantations (HTx) in patients with MCS. We aimed to investigate the effect of the new UNOS allocation system on the need for a permanent pacemaker and associated complications following HTx. Methods: The UNOS Registry was questioned, to identify patients that received HTx in the US between 2000 and 2021. The primary objectives were to identify risk factors for the need for a pacemaker implantation following HTx. Results: 49,529 HTx patients were identified, 1421 (2.9%) requiring a pacemaker post-HTx. Patients who required a pacemaker were older (53.9 ± 11.5 vs. 52.6 ± 12.8 years, *p* < 0.001), more frequently white (73% vs. 67%; *p* < 0.001) and less frequently black (18% vs. 20%; *p* < 0.001). In the pacemaker group, UNOS status 1A (46% vs. 41%; *p* < 0.001) and 1B (31% vs. 27%; *p* < 0.001) were more prevalent, and donor age was higher (34.4 ± 12.4 vs. 31.8 ± 11.5 years; *p* < 0.001). One-year survival was no different between the groups (HR: 1.08; 95% CI: 0.85, 1.37; *p* = 0.515). An era effect was observed (per year: OR: 0.97; 95% CI: 0.96, 0.98; *p* = 0.003), while ECMO pre-transplant was associated with lower risk of a pacemaker (OR: 0.41; 95% CI: 0.19, 0.86; *p* < 0.001). Conclusions: While associated with various patient and transplant characteristics, pacemaker implantation does not seem to impact one-year survival after HTx. The need for pacemaker implantation was lower in the more recent era and in patients who required ECMO pre-transplant, a finding explained by recent advances in perioperative care.

## 1. Introduction

There has been enormous progress on reducing morbidity and mortality in heart transplantation (HTx) since it was first performed by Dr. Christiaan Barnard over 50 years ago [1]. HTx remains the standard of care for selected patients with advanced heart failure (HF). In addition to graft failure, infection and stroke, sinus node dysfunction (SND) and atrioventricular block are other common complications following heart transplantation, with a reported incidence of 5–20% and 10%, respectively [2,3]. Approximately 10% of post-HTx patients require permanent pacemaker implantation, due to SND [2]. SND tends to occur early on, post HTx, and therefore most patients receive a pacemaker implantation within 30 days post HTx [2,4], compared with high-grade atrioventricular block requiring pacing later [5]. With the advent of bicaval anastomosis, the risk of SND has decreased significantly [5,6]. Increasing donor age and recipient age are also risk factors for future pacemaker implantation [6]. Varying pacing dependence after some years has been reported [5]. Nevertheless, bradyarrhythmia requiring a pacemaker has a relatively excellent long-term prognosis [6].

The 2006 United-Network-for-Organ-Sharing (UNOS) three-tiered allocation system was replaced by a system with six tiers in 2018, with a greater recognition of mechanical circulatory support [1]. As a result, more HTx takes place in those with extracorporeal-membrane-oxygenation (ECMO) or mechanical-circulatory-support (MCS) devices.

This paper discusses the potential impact of the new UNOS allocation system on the need for a pacemaker and associated complications following HTx. We performed an analysis of the UNOS database to investigate the incidence, risk factors, prognostic factors, and the relevant complications of pacemaker implantation among heart-transplant recipients.

## 2. Methods

### 2.1. Study Population

The United Network for Organ Sharing (UNOS) Registry was retrospectively reviewed to identify all patients that received a heart transplantation in the US between 2000 and 2021. Analysis of the data of UNOS Registry does not require Institutional-Review-Board approval, since it contains de-identified information of included patients.

All patients over 18 years of age who received an isolated HTx during the study period were included in this analysis. Exclusion criteria included candidates <18 years old, those undergoing simultaneous lung, liver or abdominal transplantation, and those with incomplete outcome data. The study population was divided into patients who required pacemaker implantation post-operatively, and those who did not.

### 2.2. Primary and Secondary Objectives

The primary objectives were to identify risk factors for the need of permanent pacemaker implantation following HTx.

### 2.3. Statistical Analyses

Normality for continuous variables was tested with the Shapiro–Wilk test and graphically assessed by Q-Q plots; they are presented as mean ± standard deviation (SD). Differences between groups were assessed using unpaired *t*-test. Categorical variables were expressed as frequency (%) and compared with the chi-squared test. Logistic-regression models were implemented for the identification of risk factors for post-transplant pacemaker implantation. Clinically significant factors were assessed, and the ones that were statistically significant in the univariate analysis were eventually used in the multivariable-logistic-regression model presented herein. Analyses were performed using Stata 17.0 (College Station, TX, USA). Figures were designed in Graphpad Prism 8.0 for MacOs. All tests were two-sided, and *p* < 0.05 was considered as statistically significant.

## 3. Results

### 3.1. Patient Characteristics

**Figure 1:**
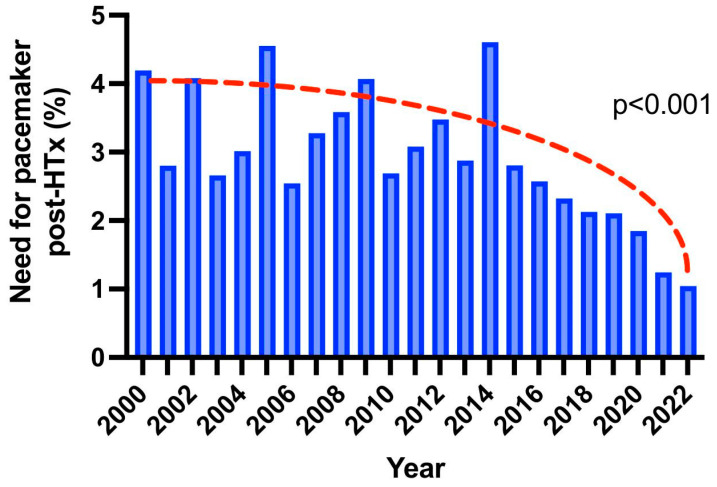
Bar plots indicating the trend of need for pacemaker implantation post heart transplantation over the years.

A total of 49,529 HTx patients were identified in the UNOS database between 1 January 2000, and 31 October 2021. Patients were stratified according to whether they underwent pacemaker implantation post transplantation (*n* = 1421; 2.9%), or not (*n* = 48,108; 97.1%) (Table 1). From 2000 through 2021, the need for post-operative pacemaker transplantation per year exhibits a downward trend, starting from 4.2% and decreasing to 1.1%, with 2020 being the first year that the incidence of pacemaker implantation was less than 2% (Figure 1).

Baseline characteristics of the study population are outlined on Table 1. Patients who required a pacemaker were older (53.9 ± 11.5 vs. 52.6 ± 12.8 years, *p* < 0.001), while male gender was predominant in both groups (76% and 74%, respectively; *p* = 0.082). More white (73% vs. 67%; *p* < 0.001) and fewer black (18% vs. 20%; *p* < 0.001) patients required a pacemaker. Cause of heart failure did not differ when divided to ischemic or non-ischemic etiology (*p* = 0.071). UNOS status 1A (46% vs. 41%; *p* < 0.001) and 1B (31% vs. 27%; *p* < 0.001) were more prevalent in the pacemaker group, while this population was also waitlisted for a longer period of time (237 ± 400 vs. 216 ± 375 days; *p* = 0.041). Donor age was higher (34.4 ± 12.4 vs. 31.8 ± 11.5 years; *p* < 0.001) in the pacemaker group, while no difference was noted in terms of ischemic time (3.3 ± 1.1 vs. 3.2 ± 1.1 h; *p* = 0.573) and distance from donor to recipient (192 ± 220 vs. 185 ± 215 miles; *p* = 0.244) (Table 1).

### 3.2. Transplantation Outcomes and Risk Factors for Pacemaker Implantation

**Figure 2:**
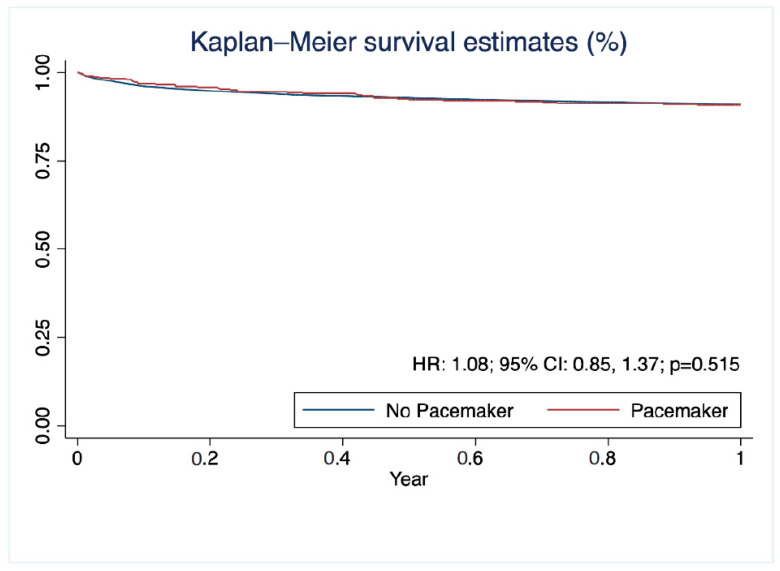
Kaplan–Meier curves showing 1-year survival post heart transplantation according to the need for pacemaker implantation.

**Figure 3:**
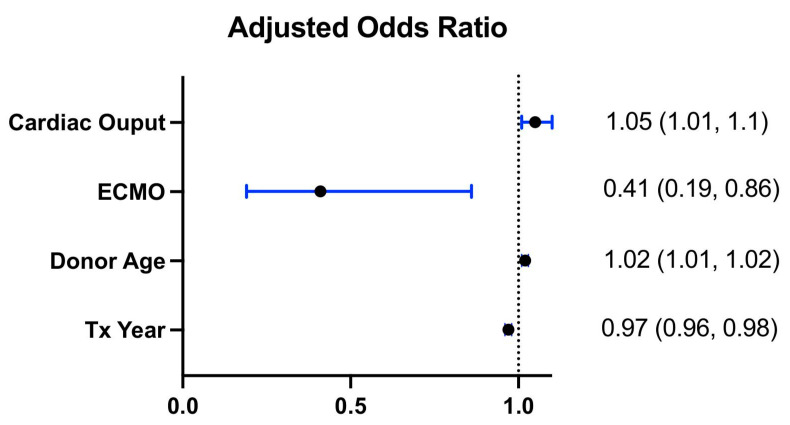
Forest plots indicating adjusted odds ratios for predictors of the need for pacemaker implantation post heart transplantation.

The 1-year survival of patients who required pacemaker implantation post transplantation did not differ from those who did not (HR: 1.08; 95% CI: 0.85, 1.37; *p* = 0.515) as shown in the Kaplan–Meier curves in Figure 2. However, there was a marginally increased risk of stroke in the postoperative period in patients requiring a pacemaker (OR: 1.30; 95% CI: 0.98, 1.73; *p* = 0.073) and a significant risk for dialysis (OR: 1.23; 95% CI: 1.06, 1.43; *p* = 0.007). Multivariable analysis for postoperative pacemaker implantation showed that cardiac output (OR: 1.05; 95% CI: 1.01, 1.10; *p* = 0.041) and donor age (OR: 1.02; 95% CI: 1.01, 1.02; *p* = 0.003) were significant risk factors. An era effect was also observed (per year: OR: 0.97; 95% CI: 0.96, 0.98; *p* = 0.003), while the need for ECMO pre-transplant was associated with a lower risk of pacemaker need (OR: 0.41; 95% CI: 0.19, 0.86; *p* < 0.001) (Figure 3).

## 4. Discussion

In this retrospective analysis of the UNOS database focusing on the incidence and risk factors for pacemaker implantation after heart transplantation, we found: (a) increasing donor and recipient age, recipient race, and status 1A and 1B association with pacemaker implantation, (b) a decreasing need for post-transplant pacemakers in the modern era, (c) no survival difference associated with pacemaker implantation at 1 year post-transplant, (d) no difference in ischemic time between the pacemaker and non-pacemaker groups, and (e) association of the need for pre-transplant ECMO with a lower incidence of pacemaker implantation.

We observed several predictors of pacemaker implantation that have been mentioned in the prior literature. In our analysis, many demographic factors such as donor and recipient age and recipient race were associated with pacemaker implantation. Cantillon et al. performed an analysis of the UNOS dataset between 1997 and 2007 [6] showing similar results, whereas Wellmann et al.’s single-center study found no relationship between pacemaker implantation and age of donor or recipient [7]. Considering the power of multi-center UNOS data pooling together with patients from various centers, in comparison to single-center studies, it is likely that there is a true relationship between donor/recipient age and pacemaker need.

Ischemic time has been previously reported to be an attributing factor [8]; however, there was no significant difference in ischemic time between the pacemaker and non-pacemaker group in our analysis of the UNOS database. Moreover, patients needing pacemaker implantation were more frequently enlisted as status 1A and 1B, and this also correlated with length of time on the waitlist. Status 1A and 1B, encompassing need for mechanical circulatory support, imply a higher severity of underlying heart disease or systemic condition of the recipient. However, our results revealed that the need for pre-transplant ECMO was related to a lower incidence of pacemaker implantation. This likely indirectly resulted from a shorter waitlist time, secondary to a more urgent heart condition requiring transplant.

The incidence of post-transplant pacemaker implantation has decreased significantly over the last two decades, based on this retrospective analysis, with an incidence of less than 2% in 2020. The trend of decrease in incidence has to be largely attributed to the bicaval-anastomosis technique, compared to the 9.1% incidence of pacemaker implantation following a biatrial anastomosis, according to a study conducted in a single center [9]. We observed an era effect in terms of a reducing incidence in pacemaker implantation. In contrast, Rivinus et al. denied an era effect, finding no relevant imbalance in pacemaker implantation from 1989 to 2018, prior to the UNOS allocation-system changes. [8] The era effect of a reducing pacemaker requirement may be explained by recent advances in perioperative care and the modification of risk factors, such as a greater utilization of the bicaval-anastomosis technique, a greater reliance on ECMO, and an improved donor-allocation system. ECMO reduces myocardial work and provides complete hemodynamic support [10], and therefore may reduce the risk of bradycardia and pacemaker necessity.

We found no survival difference between pacemaker and non-pacemaker groups at 1 year post-transplantation, consistent with several published single-center studies [7,8,11,12,13]. Of note, Wellmann et al. excluded early mortality within 3 months post transplantation, because the authors believed that patients who died early never had the chance to receive a pacemaker, and noted no long-term survival benefit [7]. In contrast, Cantillon et al.’s study using the UNOS database between 1997 and 2007 showed that pacemaker insertion was associated with an improved survival and lower five-year mortality [6]. In the early post-operative period, many factors may lead to patient mortality and serve as confounders to pacemaker insertion. As years pass, the era effect that we have observed yields a lower incidence of implantation; this may mask true differences in mortality. Prior studies have also demonstrated differential survival in early vs. late post-operative pacemaker implantation, but the limitations of our dataset prevented us from making these comparisons [8,13].

On the other hand, there was increased risk of adverse outcomes in the immediate postoperative period, such as dialysis and stroke (marginally significant) in post-heart-transplant patients who received pacemakers. No clear evidence was identified for the marginally increased risk of stroke, from our study. Acute kidney injury is a relatively common complication of heart transplantation, and could have feasibly occurred due to poor hemodynamics in the critically ill, more frequently Stage 1A and 1B patients, who required pacemaker implantation [14,15]. Survivors of acute kidney injury are subsequently at higher risk for progression to end-stage renal disease requiring dialysis [16]. Greenspon et al. found that in patients receiving a pacemaker, baseline clinical characteristics, including prior neurologic events, prior systemic embolism, age, hypertension, and New-York-Heart-Association functional class, as well as newly reported atrial fibrillation, were associated with subsequent stroke [17]. New-onset atrial fibrillation has been reported after pacemaker implantation, and could lead to an embolic neurological event [18].

There are a few limitations of the present study. First, this is a retrospective study using the UNOS database. Analysis was limited to the available information in the dataset. For example, information regarding operative timing, operative type, pacemaker indication, timing of implantation, severity of the clinical condition, and radiographic detail, was lacking. Most importantly, UNOS does not provide data on the type of anastomosis, especially for bicaval anastomosis, which largely impacts the occurrence of sinus-node dysfunction. Hence, we were unable to determine the impact of post-heart-transplant pacemaker implantation on patient functional status, quality of life, and progression of adverse events. Second, the effect of changes in UNOS allocation policy demonstrates association rather than causation, given the observational nature of the analysis. Although multiple confounding factors were adjusted in the paper, the possibility of residual confounding cannot be eliminated. Third, only limited data are available for after the change in the UNOS allocation policy in 2018. The long-term effects from the policy change have yet to be determined. The main strength of our study is that UNOS is a clinical database reflecting current practices in the United States. Analysis using the information from this database aims to improve clinical practice.

## 5. Conclusions

This retrospective study of the UNOS database examining the incidence and risk factors of pacemaker implantation after HTx found that various patient and transplant characteristics, including donor and recipient age, recipient race, and UNOS status, were associated with an increased risk of pacemaker implantation. The need for pacemaker implantation was also found to be lower in the more recent era and in patients who required ECMO pre-transplant. While there was an increased risk of stroke and dialysis in patients who received pacemakers, there was no difference in 1-year survival between pacemaker and non-pacemaker groups. These findings underscore the complex interplay of factors that contribute to the need for pacemaker implantation after HTx, and are reflective of recent advances in perioperative care. Further follow-up of these patients is necessary, to elucidate long-term differences between groups.

## Figures and Tables

**Table 1:** Patient demographics, clinical characteristics of donor and recipient populations according to the need for pacemaker implantation post heart transplantation.

Variable	Pacemaker (*n* = 1421)	No Pacemaker (*n* = 48,108)	*p*-Value
**Recipient characteristics**			
Male gender, n (%)	1080 (76)	35,377 (74)	0.082
Age, years	53.9 (11.5)	52.6 (12.8)	<0.001
Race			
White, n (%)	1032 (73)	32,281 (67)	<0.001
Black, n (%)	253 (18)	9765 (20)	
Other, n (%)	136 (9)	6062 (13)	
BMI, kg/m^2^	27.5 (4.8)	27.7 (4.7)	0.745
Cardiomyopathy type			0.071
Non-ischemic (%)	707 (50)	24,144 (50)	
Ischemic (%)	523 (37)	17,067 (35)	
Other (%)	191 (13)	6897 (15)	
UNOS status			
1A, n (%)	648 (46)	19,634 (41)	<0.001
1B, n (%)	441 (31)	13,054 (27)	
1, n (%)	12 (1)	939 (2)	
2, n (%)	82 (6)	4869 (10)	
3, n (%)	41 (3)	1830 (4)	
4, n (%)	34 (2)	2012 (4)	
ABO group			
A, n (%)	577 (41)	19,644 (41)	0.171
B, n (%)	203 (14)	7092 (15)	
AB, n (%)	83(6)	2534 (5)	
O, n (%)	557 (39)	18,808 (39)	
Creatinine at the time of transplant, mg/dL	1.3 (0.7)	1.4 (0.9)	0.129
CPRA, %	12.1 (22.5)	11.3 (23.6)	0.528
PCWP at transplant, mmHG	18.5 (8.8)	18.2 (8.8)	0.341
sPAP at transplant, mmHg	41.1 (14.8)	41.2 (14.7)	0.717
mPAP at transplant, mmHg	27.7 (10.5)	27.8 (10.5)	0.693
Cardiac output at transplant, L/min	4.6 (1.5)	4.5 (1.5)	0.063
ECMO, n (%)	4 (0.3)	659 (1.4)	<0.001
Inotropes, n (%)	511 (36)	16,519 (34)	0.204
Total time on waiting list, days	237 (400)	216 (375)	0.041
**Donor characteristics**			
Male gender, n (%)	1007 (71)	34,077 (71)	0.980
Age, years	34.4 (12.4)	31.8 (11.5)	<0.001
HCV, NAT positive, %	0 (0)	37 (0.1)	0.042
**Transplantation characteristics**			
Ischemic time, hours	3.3 (1.1)	3.2 (1.1)	0.573
Distance from donor to recipient in miles	192 (220)	185 (215)	0.244

BMI: body mass index; UNOS: United Network for Organ Sharing; CPRA: calculated panel-reactive antibody; PCWP: pulmonary capillary wedge pressure; sPAP: systolic pulmonary artery pressure; mPAP: mean pulmonary artery pressure; ECMO: extracorporeal membrane oxygenation; HCV: hepatitis C virus; NAT: nucleic acid amplification test. UNOS status: old allocation 1A: VA-ECMO, non-dischargeable BiVAD, LVAD with life threatening VT/Vfib, non-dischargeable LVAD, balloon pump, complicated LVAD course; 1B: dischargeable LVAD, on inotropes, congenital heart disease, retransplant; new allocation: 1: VA ECMO, non-dischargeable BiVAD, LVAD with life threatening VT/Vfib; 2: non-dischargeable LVAD, balloon pump; 3: complicated LVAD course; 4: dischargeable LVAD, on inotropes, congenital heart disease, retransplant.

## Data Availability

Data available in UNOS database.
